# Variability in Skin Prick Test Results Performed by Multiple Operators Depends on the Device Used

**DOI:** 10.1097/WOX.0b013e31827e6513

**Published:** 2012-12-15

**Authors:** Rebecca L Werther, Sharon Choo, Katherine J Lee, Debra Poole, Katrina J Allen, Mimi LK Tang

**Affiliations:** 1 Department of Allergy and Immunology, Royal Children's Hospital, Melbourne, Australia; 2 Murdoch Childrens Research Institute, Melbourne, Australia; 3 Department of Paediatrics, University of Melbourne, Melbourne, Australia

**Keywords:** device, skin prick test, variability

## Abstract

**Background:**

The variability of skin prick test results when carried out by multiple users has not previously been assessed across different devices or between different sites on the body. Such multiuser variability has important implications for clinical practice.

**Objectives:**

We assessed the variability of measurements from 4 commonly used single-headed skin test devices when used by multiple operators and examined whether the variability in performance was different on the back compared with the forearm.

**Methods:**

Eight adult volunteer "operators" were trained in the use of 4 devices: Greer Pick, Quintip, Stallergenes Lancet, and Feather Lancet. Each operator performed a histamine skin prick test with all devices on the backs and forearms of 5 volunteer "receivers." Variability in results was assessed using a multilevel (random effects) regression model.

**Results:**

After controlling for variation between users and receivers, the residual variability or "measurement error" was least for the Stallergenes Lancet, closely followed by the Quintip. The Greer Pick had the greatest variability. There was greater variability in measurements on the arm compared with the back.

**Conclusions:**

The devices using the "puncture" method (Stallergenes Lancet, Quintip) provide less variability in results than those using a "prick" method when carried out by multiple users (Greer Pick and Feather Lancet). Testing on the back also gives less variable results compared with the arm.

## Introduction

Skin prick testing (SPT) is the primary investigation used by allergists to aid in the diagnosis of immunoglobulin E-mediated allergy [1]. Although the doctor-supervised oral food challenge is the gold standard for the diagnosis of food allergy, a detailed medical history together with SPT can help to determine the likelihood that one is allergic to a food and subsequently guide further management [2,3]. Furthermore, serial SPT over time is often used to monitor for resolution of food allergy, providing clinical decision points for when an oral food challenge may be of assistance in assessing for acquisition of tolerance. It is therefore important that SPT can offer reproducible results with high precision and accuracy. In busy clinical practices and large population-based epidemiologic research studies, a number of operators often perform SPT and it is important that the results across the multiple operators and the multiple patients are reproducible. Both clinical and research settings would benefit from use of an SPT device that offered low variability when administered by multiple users, as this would allow greater reproducibility (precision and accuracy) of SPT results.

Currently, several single-headed SPT devices are used within specialist allergy centers internationally. Several articles have compared the variability in the measurements for different devices when used by a single operator (intrauser variability)[4-6]; however, no study to date has assessed variability in performance of devices used by different operators (multiuser variability). Variability of SPT results performed by a single operator on the back compared with the arm has previously been assessed[4]; however, multiuser variability of testing performed on the arm versus back has not been compared for different SPT devices.

The aim of this study was to compare the variability of skin test results from 4 commonly used single-headed SPT devices when carried out by multiple operators and to compare the variability in results when SPT tests are carried out on the back compared with the forearm.

## Materials and Methods

### Study Design

Using a prospective study design, 5 healthy adult volunteer subjects received SPTs on the forearm and the back with each of 4 devices, performed by 8 different operators. The study was reviewed and approved by the Royal Children's Hospital Human Research Ethics Committee. All subjects enrolled into the study gave signed informed consent.

### Inclusion and Exclusion Criteria

Healthy volunteers older than 18 years were recruited by poster advertising within the hospital to be SPT "operators" or "receivers." The operators consisted of adult staff members of various backgrounds, including medical and clerical staff, statisticians, and scientists. Novice operators were chosen to standardize the level of training of each operator with each device, with a consistent level of training for each of the 4 devices. Volunteers were therefore ineligible as operators if they had previously worked within a clinical allergy service or used any of the SPT devices. Receivers did not take antihistamines 1 week before testing. Eight operators and 5 receivers were recruited into this study (although 1 receiver withdrew and was replaced midway through the study).

### Devices

**Figure 1:**
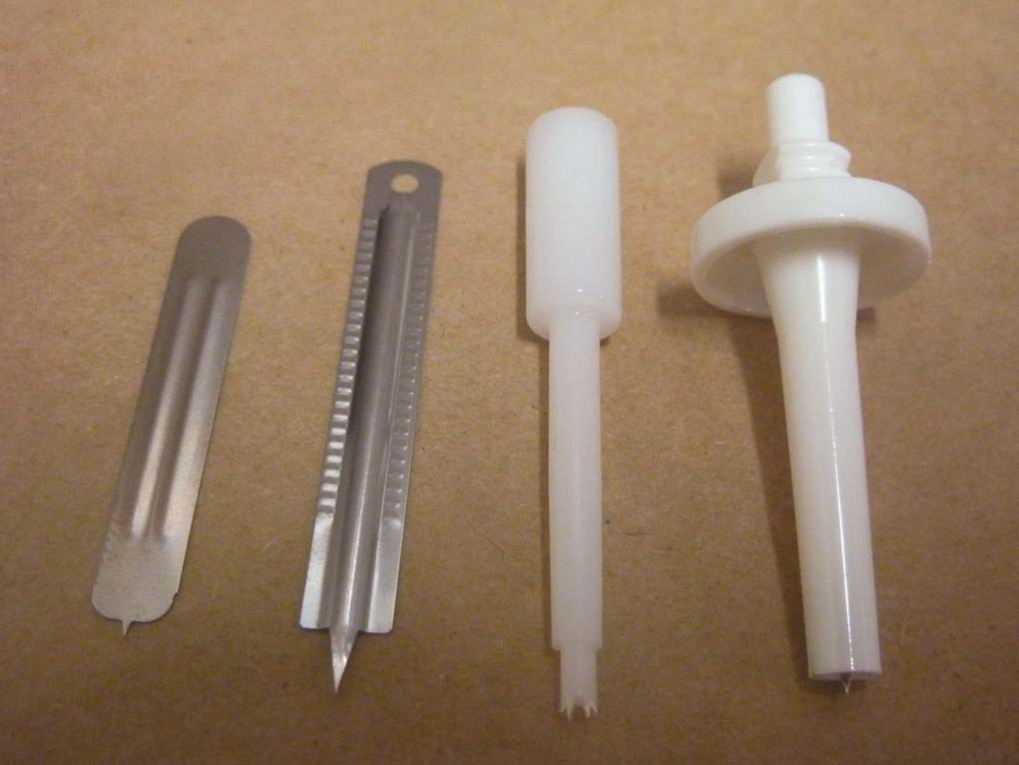
**Skin prick test devices investigated (left to right): Stallergenes Lancet, Feather Lancet, Greer Pick, and Quintip**.

Four different devices were chosen based on performance in published comparative studies and clinical use. The Greer Pick (previously known as "DermaPik"; Greer Laboratories, Lenoir, NC) and Quintip (Hollister-Stier Laboratories, Spokane, WA) were previously reported to perform well when used by a single operator, with low variability in results and high sensitivity and specificity [4,5]. The Greer Pick and Quintip were also previously reported to have the lowest and second lowest pain scores of 8 devices tested, respectively [4]. The Stallergenes Lancet (Stallergenes, Antony Cedex, France) and the Feather Lancet (Graham Field Health Products, Atlanta, GA) were selected as they have been used in published studies assessing positive predictive values for presence of food allergy [7,8] and are commonly used in clinical practice (Figure 1).

All 4 devices were used according to the manufacturer's instructions. Quintip and Stallergenes Lancet were applied by the "puncture method" (device applied at 90-degree angle with downward pressure). Feather Lancet and Greer Pick were applied by the "prick method" (applied at 45-degree angle with slight lifting on withdrawal) [9]. Devices were used once and then discarded.

### Training

Operators were trained to use the 4 devices by a senior allergy nurse and allergy physician over a 2-week period. Both the nurse and the physician had been trained either by representatives of the device manufacturing company or by other senior allergy clinicians experienced with these devices. Training consisted of 2 × 4-hour sessions, with 2 hours dedicated to teaching and practicing each device. Volunteers practiced on each other with both saline and histamine under supervision and guidance by the training doctor and nurse. Logbooks were kept by each operator, and attainment of training was defined as having completed at least 100 SPTs using each device. Proficiency in SPT technique was defined as demonstrating correct technique when performing 5 consecutive histamine SPTs for each device under supervision by the training nurse and physician. The training was deemed to be similar to the amount a new nurse or clinician would receive before performing skin tests on patients.

### Comparison of SPT Devices

Each operator performed a single SPT with each device on each receiver on both the arm (32 SPTs on each receiver's arm) and the back (32 SPTs on each receiver's back). Thus, a total of 320 skin prick measurements (5 receivers × 32 measurements × 2 locations) were available for analysis. All SPTs on the backs were carried out in a single session, and all SPTs on the arms were carried out in another single session 1 week later. Both sessions were performed at the same time in the afternoon to avoid diurnal variation in skin reactivity. All SPTs were performed using histamine (1 mg/mL; Hollister-Stier) to compare variability of skin test size using a "positive" control. SPTs were spaced a minimum of 2 cm apart. SPTs on the back were performed on the upper back, with each person's upper back divided into 4 quadrants and the 8 SPTs for each device performed in a single quadrant. SPTs on the arms were performed on the volar aspect of the forearm. Each forearm was divided into 2 sections, with 8 SPTs for each device in a single section.

Wheal sizes were measured after 10 minutes [10] by a single senior allergy nurse, and results were recorded as the mean of the largest and perpendicular diameters.

### Statistical Methods

Results are summarized by device and location for all observations, including the number of false-negative results (wheal size < 3 mm), presumably representing an error due to user technique, and the number of 0 mm results, representing failure or "mis-fire" of SPT. A formal comparison of the variability in wheal size for each of the 4 devices was performed using a single multilevel (random effects) model. This model allowed for different average wheal size by device and location (included as fixed effects) and allowed for correlations between observations taken by the same operator and carried out on the same receiver using random effects. The model separates the variability in SPT measurements into 3 components: variability between operators, variability between receivers, and variability within operators and receivers, known as "random" or "measurement" error. The measurement error represents variability in SPT measurements caused by unpredictable fluctuations in the readings from the devices, which are not explainable by differences in who carried out or received the test, and hence are reflective of variability within the actual device used to perform the test. The outcome of interest was the overall variability in measurements, which is the sum of these 3 components of variability.

To determine whether the unpredictable fluctuations were different for the 4 devices and between measurements taken on the arms and back, we applied a single regression model with 8 error terms, allowing a different amount of unpredictable fluctuation for each device and location combination.

Similarly, to assess whether the variability between operators and between receivers was different across the 4 devices and between measurements taken on the arms and back, we used 8 random effects to model the between-operator variability, allowing a separate estimate of the variability between operators for each device and location combination, and 8 random effects to model the between-receiver variability, allowing a separate estimate of the variability between receivers for each device and location combination.

Results from the best fitting model, combining variance estimates across devices/locations where there was no evidence that these differed, are presented. Model fit was compared using the Bayesian Information Criteria, a measure of goodness of fit that takes into account the fit of the model and its complexity. Smaller values of the Bayesian Information Criteria represent better model fit, with differences of > 3 between results indicative of a better model fit. Results are expressed as estimates of variability both within (measurement error) and between receivers and operators.

A sample size calculation could not be performed due to the complexity of the statistical analysis; hence, the number of participants was chosen based on feasibility.

## Results

### Variability of SPT Wheal Size by Device

**Figure 2:**
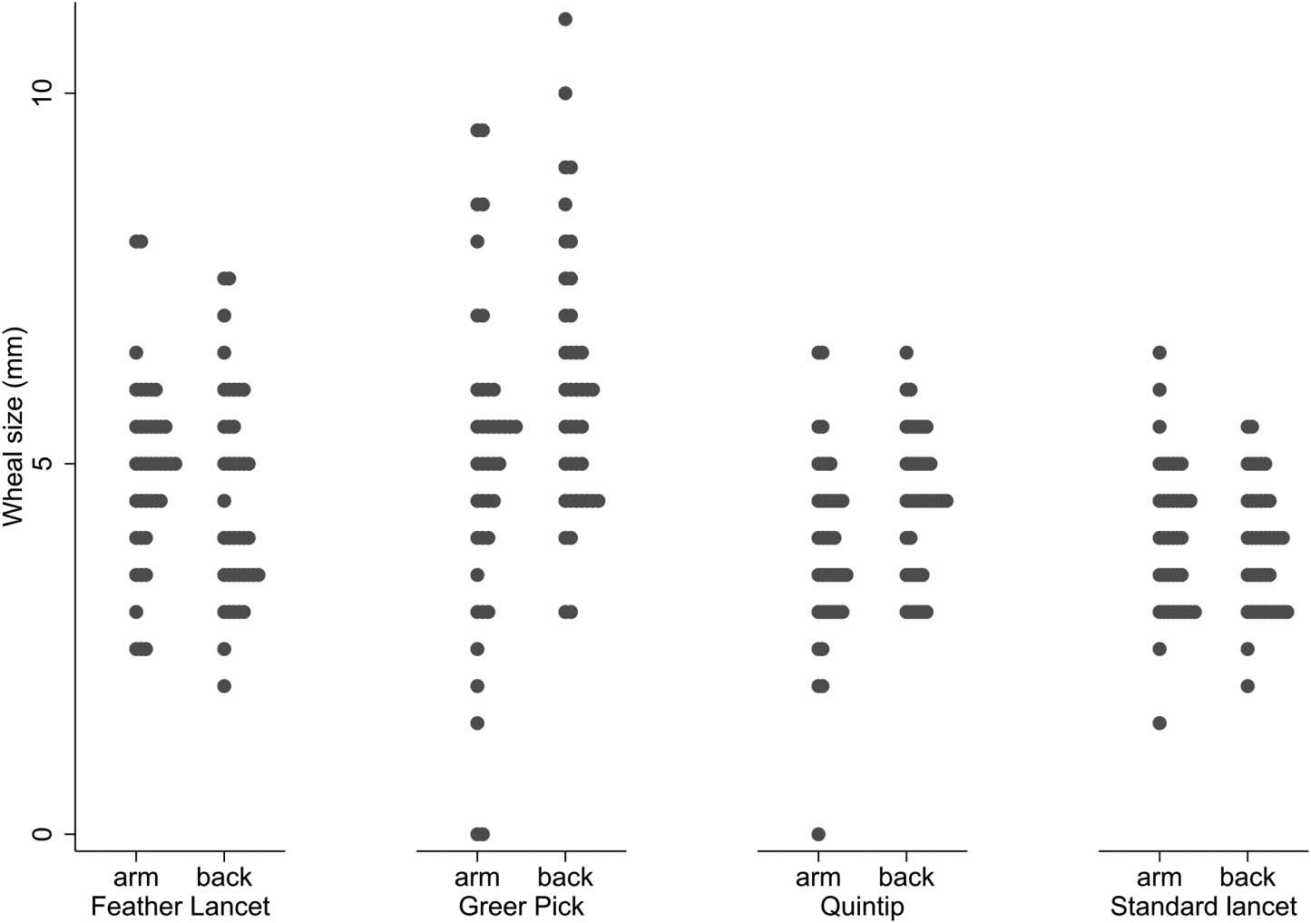
**Wheal size by device and location**. Comparison of wheal size (millimeters) results between each device on the arm and the back. Each dot represents a single skin test result. Width of bands is indicative of number of dots at that wheal size.

Table 1 and Figure 2 show summaries of the raw data for all SPTs. From these raw data, it seemed that SPT measurements obtained using the Greer Pick were the most variable, whereas measurements from the Stallergenes Lancet were the least variable. The Quintip and Greer Pick were the only devices that had test "failures," with similar numbers of false-negative results (< 3 mm) across the 4 devices.

**Table 1:** Summary of Wheal Size by Device and Location (n = 40 for Each Device by Location)

Device	Location	Mean, mm	SD, mm	No. Results < 3 mm (Including 0 mm), %	No. Results = 0 mm, %
Feather Lancet	Arm	4.90	1.25	3 (7.5)	0 (0)
	Back	4.53	139	2 (5)	0 (0)
Greer Pick	Arm	4.99	2.17	5 (12.5)	2 (5)
	Back	6.05	1.81	0 (0)	0 (0)
Quintip	Arm	3.84	1.23	5 (12.5)	1 (2.5)
	Back	4.49	0.95	0 (0)	0 (0)
Stallergenes Lancet	Arm	4.00	1.01	2 (5)	0 (0)
	Back	3.86	0.85	2 (5)	0 (0)

Exploring the variability of measurements from the SPT devices using multilevel modeling found that the most appropriate model allowed the measurement error term to vary by device and location (ie, fitting 8 error terms) but the between-receiver and between-operator variability to vary only by location (ie, 2 random effects for receiver and 2 random effects for operators). The results from this best fitting model are presented in Table 2. These data suggest that the measurement error was smallest with the Stallergenes Lancet, closely followed by the Quintip and the Feather Lancet, whereas the measurement error was largest with the Greer Pick. There was little evidence that the variability between operators or receivers varied across devices, meaning that the differences in the overall variability in measurements between the 4 devices were driven by the measurement error of the devices. Of particular interest, there was evidence that the measurement error (and therefore the overall variability) was larger for all devices (Quintip, Greer Pick, and Feather Lancet) when compared with the "gold standard" Stallergenes Lancet (P value from the likelihood ratio test comparing the pooled variability across the 3 devices with that from the Stallergenes Lancet < 0.001).

**Table 2:** Variability of Skin Prick Test Measurements by Device and Location

Device	Arm	Back
Stallergenes Lancet random error	0.43 (0.28-0.76)	0.47 (0.30-0.82)
Quintip random error	1.01 (0.66-1.74)	0.62 (0.40-1.08)
Feather Lancet random error	1.02 (0.66-1.77)	1.23 (0.83-2.18)
Greer Pick random error	3.55 (2.37-5.91)	2.27 (1.50-3.84)
All devices		
Between-tester variability	0.16 (0.06-1.20)	0.26 (0.10-1.50)
Between-receiver variability	0.55 (0.19-5.31)	0.18 (0.06-2.48)

Estimates of variability (and 95% confidence intervals) from a single multilevel/random effects model allowing for variability between testers and receivers and random measurement error. Larger numbers reflect greater levels of variability.

### Variability of Wheal Size When SPT Was Performed on the Back Versus Arm

There was more variability between receivers when measurements were taken from the arm compared with the back (estimate of variability = 0.55 for arms compared with 0.18 for the back). In contrast, there was less variability between operators for measurements on the arm compared with the back. This partially counterbalances the greater variability in measurements taken from the arms compared with the back.

There were larger measurement errors for the Greer Pick and the Quintip in SPTs carried out on the arm compared with the back, with little difference in measurement error with Stallergenes Lancet, and slightly smaller measurement error on the arm compared with the back with the Feather Lancet.

When considering all of the variability elements together, the relatively large variability between receivers in arm measurements resulted in the overall variability being larger in measurements taken on the arm compared with the back.

## Discussion

We performed a prospective study to compare the variability in wheal size from SPTs carried out using 4 commonly used devices when carried out by multiple operators. The performance was assessed by comparing the overall variability in measurements taking into account the between-operator variability, the variability between receivers, and random measurement error of SPT results. Variability in SPT results was also compared between SPTs carried out on the arm and the back. Importantly, this is the first study to compare the variability of these 4 devices when carried out by multiple operators and in particular the first study to assess the between-user variability of the SPT devices. It is also the first study comparing SPT on the arm and the back using various single-headed devices and more than 1 operator. The results presented in this article identify clear differences in the performance of the devices; after controlling for variation between operators and receivers, the residual variability or "measurement error" was least for the Stallergenes Lancet, closely followed by the Quintip, whereas the Greer Pick had the greatest measurement error.

SPTs have previously been compared when performed multiple times by a single operator [4-6]. Quintip and Greer Pick were shown to have 95 and 98% sensitivity, respectively, with 100% specificity for both the devices [4]. The previous studies show which device is most accurate if skin testing is performed by the same clinician on each occasion but has limited relevance in situations where testing is carried out by multiple operators. In many clinical and research settings, skin testing is performed by more than 1 operator. To accurately interpret results of SPT in these settings, it is important for the SPT to offer a low-level variability when performed by multiple users. There are several other variables that can influence the wheal size when performing SPT [11] such as extract quality, time of day, location on the skin, and the measuring of results. In our study, we have accounted for these by using the same positive control for all subjects and by performing the skin tests within the same region of the body and at the same time of day. Furthermore, all SPT wheals were measured by the same experienced allergy nurse.

As part of this study, we also compared the variability of SPTs carried out on the forearm versus the back. The results suggest that measurements taken from the arm are more variable than measurements taken from the back, with larger between-receiver variability in the arm measurements. As there are no studies comparing the variability between single-headed devices across the 2 anatomical sites when carried out by multiple operators, our study provides the first evidence regarding which anatomical site offers more accurate SPT results.

We used histamine, rather than a specific allergen, for all SPTs as it should cause a wheal of ≥ 3 mm to develop with every skin test on all recipients. Due to the expected variation in histamine response between recipients, we focused on the variability of wheal sizes rather than the actual size of the wheal, which may be subject specific. The Greer Pick and Quintip were the only devices that had results of 0 mm (occurring only in SPTs on the arm). Although the number of 0 mm measurements is small, it suggests that these devices are more likely to result in failure to accurately perform the test on the arm. Of note, the number of false-negative results (< 3 mm) was fairly similar across all 4 devices, with more false-negative results on the arms than on the backs. This may suggest that skin testing is more accurate on the backs, not only due to reduced variability when carried out by multiple operators but also due to reduced numbers of false-negative results. A larger study would be necessary to confirm these findings regarding the rates of false-negative and failed test performance.

We found that the Quintip and Stallergenes Lancet, which are applied by the puncture method, had lower variability compared with the 2 devices administered by the "prick" method (ie, Greer Pick and Feather Lancet). Possible reasons for this difference may be the angle used to approach the skin and/or the amount of force used when collecting and pricking the superficial layer of the skin. Variability with the prick method may be reduced with more extensive training and regular monitoring and assessment of technicians; however, additional studies with larger groups and varying levels of training would be required to assess whether this was the case.

A limitation of this study is the small number of operators and receivers. Nevertheless, we included a larger number of operators than previous studies comparing SPT devices, which only assessed 1 operator [4-6]. Moreover, we were limited by the number of tests that could be performed on 1 receiver at the same time. Another limitation is the generalizability of the results. This study includes healthy adult volunteers, leaving some question as to whether these results can be generalized to adults with allergies and children.

In summary, our results show that after adjusting for variation between operators and receivers, measurements taken with the Stallergenes Lancet had the lowest variability, closely followed by Quintip, suggesting that devices applied using a puncture method may offer more reliable results than devices applied by a prick method. Second, we found that variability was greater for SPTs on the arm compared with the back. These findings have important implications for SPTs in allergy practices where several operators perform the tests and in research studies performing SPTs in large sample populations.

## End Note

Presented at Australasian Society of Clinical Immunology and Allergy Annual Meeting, September 7-9, 2011, Sydney, Australia (Poster Presentation).

## Competing interests

The authors declare that they have no competing interests.
